# *Cakile maritima*: A Halophyte Model to Study Salt Tolerance Mechanisms and Potential Useful Crop for Sustainable Saline Agriculture in the Context of Climate Change

**DOI:** 10.3390/plants13202880

**Published:** 2024-10-15

**Authors:** Ricardo Mir, Diana M. Mircea, Mario X. Ruiz-González, Paco Brocal-Rubio, Monica Boscaiu, Oscar Vicente

**Affiliations:** 1 Institute for the Conservation and Improvement of Valencian Agrodiversity (COMAV), Universitat Politècnica de València, Camino de Vera s/n, 46022 Valencia, Spain; maruigon@upvnet.upv.es; 2 Mediterranean Agroforestry Institute (IAM), Universitat Politècnica de València, Camino de Vera s/n, 46022 Valencia, Spain; dmmircea@upvnet.upv.es (D.M.M.); mobosnea@eaf.upv.es (M.B.); 3 Independent Researcher, Cook, 46530 Puzol, Spain; pacobrocalrubio@hotmail.com

**Keywords:** sea rocket, salinity, biosaline agriculture, halophyte, NaCl, antioxidant, ion transport

## Abstract

Salinity is an increasing problem for agriculture. Most plant species tolerate low or, at best, moderate soil salinities. However, a small (<1%) proportion of species, termed halophytes, can survive and complete their life cycle in natural habitats with salinities equivalent to 200 mM NaCl or more. *Cakile maritima* is a succulent annual halophyte belonging to the Brassicaceae family; it is dispersed worldwide and mainly grows in foreshores. *Cakile maritima* growth is optimal under slight (i.e., 100 mM NaCl) saline conditions, measured by biomass and seed production. Higher salt concentrations, up to 500 mM NaCl, significantly impact its growth but do not compromise its survival. *Cakile maritima* alleviates sodium toxicity through different strategies, including anatomical and morphological adaptations, ion transport regulation, biosynthesis of osmolytes, and activation of antioxidative mechanisms. The species is potentially useful as a cash crop for the so-called biosaline agriculture due to its production of secondary metabolites of medical and nutritional interest and the high oil accumulation in its seeds. In this review, we highlight the relevance of this species as a model for studying the basic mechanisms of salt tolerance and for sustainable biosaline agriculture in the context of soil salination and climate change.

## 1. Introduction

### 1.1. Halophytes and Biosaline Agriculture

Soil salinity refers to the concentration of inorganic solutes in the soil aqueous phase, including soluble charged species, non-ionic solutes, and ions that combine to form ion pairs. Since sodium cations are the most damaging to plant development, from an agricultural point of view, salt-affected soils are classified as saline, sodic, and saline-sodic soils [1]. In nature, salts accumulate in soils and water mainly due to the erosion and weathering of superficial rocks, seawater intrusion in coastal areas, and rising saline aquifers [1]. The natural increase of salt amounts in soils and water is referred to as primary salinisation, resulting in areas rarely used for agriculture because of the relative sensitivity of all major crops to salinity. Moreover, many traditional agricultural areas, mainly those cultivated under irrigation in arid and semiarid regions, are affected by the so-called secondary salinisation provoked by the accumulation in the soil of ions dissolved in irrigation water but also by improper agricultural practices, such as the abuse of amendments and agrochemicals (pesticides, herbicides, fertilisers) in soil and water, and uncontrolled farming wastes, amongst others [1].

Salinisation, either primary or natural or secondary or anthropogenic, entails considerable growth and viability limitations for most plant species, including all major crops. Moreover, the increase of salts in soils alters their structure, chemistry and biology, indirectly affecting plant performance [2]. The rise of global Earth temperature and the intensification of agricultural practices increase salinity in cultivated areas, thus presenting an expanding problem for producing food and other plant-derived goods. According to the Global Map of Salt-Affected Soils (GSASmap; Ref. [3]), over 3% and 6% of global land top soils and subsoils, respectively, are affected by salinity or sodicity, arid and semiarid climatic zones being the most affected [3]. Thus, the present and future of the conventional agriculture model must be revisited to guarantee the availability of natural resources and food for future generations. In this sense, the scientific community has made a considerable effort to obtain new crop varieties with better performance under adverse environmental conditions, including salinity [4]. However, there has been limited success in the obtention of new crop varieties resistant to salinity despite the deep understanding of plant genetic and physiological responses to salt, probably because salinity has not been a primary trait for breeders and affects localised agricultural areas [5].

Intraspecific variation has been successfully used to obtain several salt-tolerant rice cultivars [6,7], whereas results are more discrete in wheat [5]. Traditional breeding programmes based on the hybridisation of commercial tomatoes with wild relatives such as *Solanum pennellii* resulted in the generation of lines with improved performance under salinity conditions [8]. Similarly, introgression of *Nax2* genes from an ancestral wheat relative (*Triticum monococcum*) into commercial durum wheat resulted in a lower sodium accumulation in shoots, thus in a greater performance under saline conditions with no apparent growth penalties [9]. The use of salinity-resistant rootstocks with more efficient sodium and/or chloride exclusion mechanisms has been tested for woody crops such as apples [10], citrus [11], or grapes [12], although further work is required to reach commercial application [5]. Similarly, salinity resistance in tomatoes has been achieved using Na^+^ exclusion tomato and eggplant rootstocks [13,14]. Finally, transgenic japonica rice plants overexpressing *OsNHX1*, the vacuolar Na^+^/H^+^ antiporter, responded better than control plants when growing under mild saline conditions [15]. All these are some examples of the relative success of the obtention of improved crops resistant to salinity. However, a proper and suitable breeding approach has not yet been reported to generate salt-resistant crops with competent agronomic features.

In recent years, the use of salinity-resistant plant growth-promoting rhizobacteria (PGPR) has emerged as an attractive possibility to mitigate the salinity problem in agriculture. Such microorganisms synthesise beneficial secondary metabolites and siderophores, as well as hormones and enzymes, and improve soil features, including ion homeostasis and nutrient and water availability, that are beneficial for plant performance. The interaction between plants and PGPR improves critical physiological processes and enhances photosynthesis efficiency, which promotes plant growth and development, especially under saline conditions [16]. Thus, the application of halotolerant PGPR inoculates in the field opens a new sustainable approach to increasing food production. As a complementary strategy to the genetic improvement of crop salt tolerance, through classical breeding and genetic engineering or NGTs (new genomic techniques) such as genome editing, as well as the application of PGPR inoculums, resilient wild plant species adapted to extreme environmental conditions in their natural habitats can be used for agricultural purposes; they can alleviate the pressure exerted by agricultural activity on the natural environment and contribute to the maintenance of cultivated lands. In the case of saline soils, the commercial cultivation of plant species adapted to saline conditions, the halophytes, represents the basis of the so-called (bio)saline agriculture [17]. Moreover, the study of halophyte biology will help us understand the strategies followed by these species to tolerate high salinity conditions [18], which could be translated, to some degree, to our conventional crops, which are glycophytes or salt-susceptible species.

### 1.2. Definition of Halophytes

Halophytes are a limited group (less than 1% of the world flora) of plant species that present a natural resistance and/or tolerance to salinity. These can be either obligate halophytes, which require constant saline environments for optimum growth, or facultative halophytes, which can withstand a certain degree of salinity, although they perform better under non-saline or low salinity conditions. Nonetheless, the precise classification of particular species as halophytes is still under debate, and the exact number of halophytes is difficult to establish. The most generally accepted definition for halophytes considers these species as plants able to grow and complete their life cycle under 200 mM NaCl or higher salt concentrations [19]. Other authors, however, established a salt concentration threshold down to 85 mM NaCl to consider a species to be a halophyte [20]; according to this criterion, a total number of ca. 6000 species, including terrestrial and marine plants, could be considered as halophytes [20]. A broader definition considers halophytes as species that can naturally grow and complete their life cycle on saline soils where most plant species cannot [21]. A less restrictive list of worldwide halophytes that includes miohalophytes or species with the best performance under non-saline conditions that tolerate low salinity (i.e., 20–100 mM NaCl) resulted in 26,000 species [22]. Aronson defined 1560 salt-tolerant plant species, selected by their ability to grow under electrical conductivity levels of 7.8 dS m^−1^, equivalent to 80 mM NaCl, during significant life cycle periods [23]. Moreover, halophytes are distributed within many plant families. From a list of only 1861 halophytes, a total of 139 families and 636 genera were identified [22]. Altogether, the exact definition and classification of halophytes is complex since salinity responses depend on many different factors, such as the soil and water salt concentration, the physiological stage of plants when salt is present, or the exposure interval. A more detailed discussion of halophyte definition and classification can be found in [24].

Finally, the web repository eHALOPH (https://ehaloph.uc.pt/ (accessed on 10 July 2024)) compiles more than 1200 species with a certain degree of natural salt tolerance, including strict halophytes that perform better under mild saline conditions (e.g., 100 mM NaCl) and can grow in the presence of 200 mM NaCl (20 dS m^−1^) or higher salinity levels, and species that tolerate salt concentrations of ~80 mM NaCl (7.8 dS m^−1^). Moreover, eHALOPH includes relevant published information about their morphological, physiological and biochemical adaptations to salinity, natural habitats, and reported economic uses [25], which is helpful for the halophyte biologist community.

### 1.3. Halophyte Responses to High Salinity

Salt cellular response triggers the activation of an early signalling pathway, including the production of Reactive Oxygen Species (ROS) and Ca^2+^ waves, and a later mechanism to avoid the toxic effects of Na^+^ cations within cells. Sodium cellular perception provokes a rapid production of ROS that results in oxidative damage, characterised by alterations in redox homeostasis, lipid peroxidation, protein oxidation, and enzymatic inhibition [18]. Moreover, calcium accumulates in the cytosol, and calcium peaks are sensed by calcineurin B-like (CBLs) Ca^2+^ interacting proteins. Upon Ca^2+^ sensing, CBLs interact with CBL-interacting protein kinases that activate the phosphorylation of target proteins [26]. Later, sodium accumulation within cells causes an osmotic imbalance that is lethal for cells. This osmotic deregulation can be alleviated by the activity of proton pumps that promote sodium cell extrusion or sequestration within vacuoles [26].

How plants, and specifically halophytes, deal with sodium in soils depends on several built-in, non-exclusive mechanisms that rely on anatomical, physiological, biochemical, and genetic adaptations. Both halophytes and glycophytes share common response mechanisms, although halophytes’ cellular and enzymatic machinery is generally more effective for sodium detoxification [27]. One of the primary defence mechanisms under high salinity conditions is sodium extrusion through the Salt Overly Sensitive (SOS) pathway. Briefly, SOS3 is a calcium-binding protein that interacts with and activates the serine/threonine protein kinase SOS2 in response to high calcium peaks provoked by sodium accumulation within cells [28]. This interaction further leads to the phosphorylation and activation of SOS1, the plasma membrane Na^+^/H^+^ antiporter that actively transports sodium from the cytosol to the apoplast [29]. Although this response pathway is shared in both halophytes and glycophytes, it is more effective in the salt-resistant *Eutrema salsugineum* and *Schrenkiella parvula* than in their relative *Arabidopsis thaliana* [30]. Whether this is a general trend, although likely, is still unresolved.

Moreover, halophytes present an efficient antioxidant machinery that reduces the cellular damage caused by the rapid increase of ROS in the cytosol. Antioxidant response in plants can be enzymatic or non-enzymatic. Enzymes such as superoxide dismutase (SOD), catalase (CAT), dehydroascorbate reductase (DHAR), and ascorbate peroxidase (APX), amongst others, can metabolise toxic ROS into non-toxic products and water. On the other hand, non-enzymatic scavenging mechanisms exist in plants and are especially important to avoid the accumulation of ^1^O_2_ and OH∙ since these ROS cannot be enzymatically catabolised [31]. The basis of the non-enzymatic system relies upon the accumulation of antioxidant compounds such as ascorbate and glutathione, glycine-betaine, and proline, amongst others, which can scavenge ROS molecules [31]. Although ROS degradation and scavenging alleviate the toxic effects of ROS, these are not the primary mechanisms that allow strict halophytes to grow under high salinity conditions. Instead, such highly salt-tolerant species are able to maintain a low cellular sodium concentration, thus low ROS and oxidative stress, from the initial salinity perception [31].

In addition, halophytes have evolved specific anatomical adaptations, such as succulent leaves or stems, and thicker epidermal layers under salt stress conditions [32,33,34], sunken stomata, tissue lignification [35], or short root systems [18]. Some halophytes have developed specialised salt-secreting structures that either directly excrete salt from the inner to the outer part of the leaves (salt glands) or temporarily accumulate salt in cavities on the surface of the leaves (salt bladders) [35].

Different halophytes show one or more of these features that allow them to handle salinity. According to the main mechanisms used by a particular species, they can be divided into three categories: *(i)* excluders or species that deal with salinity through salt avoidance, typically due to root anatomical or morphological adaptations; *(ii)* accumulators or includers, which accumulate salt within cells and tissues but avoid its deleterious effects through cellular and biochemical responses, and *(iii)* conductors, which present specialised leaf glands able to excrete salt from the inner tissues to the leaf surface [36].

In this review, we compiled reported information on *Cakile maritima*, an obligate halophyte widely spread worldwide, including all five continents, regarding the species’ biological, genetic, and ecological features, its responses to salinity, and potential use as a model for basic research on the mechanisms of salt tolerance. We also discuss *C. maritima*’s economic value as a candidate cash crop for biosaline agriculture, with diverse applications as gourmet food, oilseed crop, medicinal plant, or for the production of compounds of industrial interest, as well as its potential for desalination of salinised land and phytoremediation of heavy metal contaminated soils.

## 2. *Cakile maritima* Features

**Figure 1:**
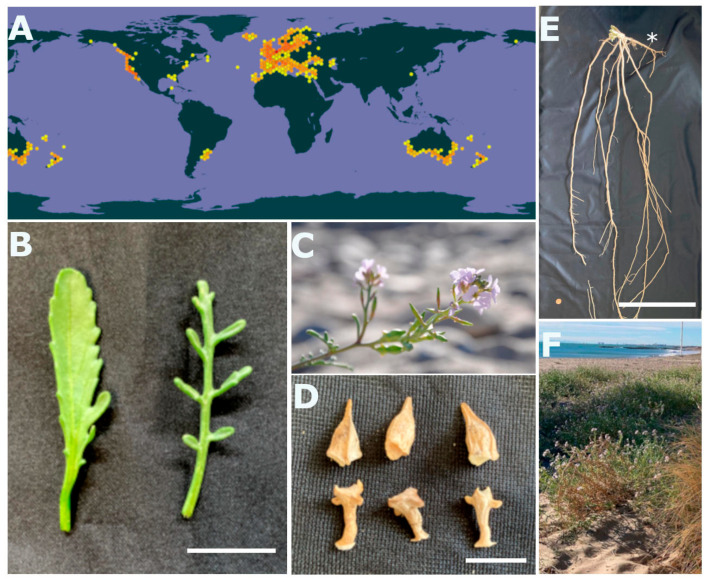
General features of *Cakile maritima*. (**A**) Natural worldwide distribution of *Cakile maritima* (extracted from (eHALOPH; Ref. [25]). Details of (**B**) leaf morphotypes, (**C**) flowers, (**D**) two-segmented dehiscent siliques, and (**E**) root (indicated with an asterisk) and horizontal stems. (**F**) Naturally growing *Cakile maritima* plants on the Mediterranean coast of Xilxes, Castellón (Spain). Scale bars in (**B**,**D**,**E**) represent lengths of 10 cm, 1 cm, and 100 cm, respectively.

*Cakile maritima* Scop. (sea rocket) is an annual European species, widely distributed along the coasts of all five continents (Figure 1A), that belongs to the Brassicaceae family. The plants are not taller than ca. 40 cm, are highly branched and can cover areas of up to one meter in diameter (Figure 1F). Leaves are glabrous and succulent and can be either oblate, oblanceolate or pinnately lobed (Figure 1B; Refs. [37,38]). The flowers are up to 25 mm in diameter, have four white to purple petals, and are highly aromatic (Figure 1C). Cross-pollination is mostly mediated by different types of insects, whereas self-pollination is rare. Fruits are green and soft when immature, blackish and lignified at maturity, and contain high amounts of lipids. The fruits consist of two joined siliquas; the upper one (terminal) is easily detached from the plant and can float and survive up to one year on saline water, favouring dispersion, whereas the bottom siliqua (proximal) usually remains in the mother plant area, thus maintaining local populations (Figure 1D; Refs. [39,40]). The main root is pivotal and can measure up to 40 cm (Figure 1E), producing many horizontal stems (Figure 1E). *Cakile maritima* is a diploid species (2n = 18) with a genome size of 719 Mb; the plants have a relatively small size, a fast life cycle (ca. three months, from seed to seed), and a high seed production rate (10,000 seeds per plant) [41,42].

*Cakile maritima* is adapted to a wide range of climate areas, from semi-desertic to arctic zones, indicating a high resistance to either heat and drought or to low temperatures and frost. Ephemeral populations are found almost exclusively in coastal regions characterised by nutrient-poor soils, intermittent increases of soil salinity due to seawater intrusion, high wind force and salt sprays over the plants’ aerial parts originating from the bursting of bubbles in breaking waves. Sandy coastal soils are mobile, have low water retention capacity, and their general nutritional value is low and dependent on the deposition of detritus. Thus, *C. maritima* is a resilient species that can grow in relatively poor soils under different climates. In particular, *C. maritima* requires nitrogen in soils but no other macronutrient for optimal photosynthesis and growth [43]. Moreover, small amounts of organic matter and slightly basic soils due to CaCO_3_ derived from shelly debris benefit sea rocket growth [40]. Altogether, the environmental and nutritional requirements of *C. maritima* are low, making this species ideal as a crop, especially in marginal lands, as proposed for other halophytes [44].

Genetic distances amongst *C. maritima* individuals of different regions in Europe are high, as shown by random amplified polymorphic DNAs (RAPDs) and Inter-Simple Sequence Repeats (ISSRs) molecular markers [45]. Genetic variability was further determined using allozyme marker-based genetic analysis on different sea rocket Tunisian populations [37]. The high genetic variability amongst *C. maritima* accessions can be explained by the low self-pollination rate and its capability to cross-pollinate with its relative *Cakile edentula* [46] and the high dispersion rate by sea currents [37]. This genetic variability explains *C. maritima* plasticity and adaptability to different environments. Indeed, *C. maritima* variants collected from different climate regions in Tunisia showed different morphological features, such as silique mass and leaf shape [47], and differences in biochemical responses to salinity. Under control conditions, plant dry weight is generally greater for humid-adapted accessions. However, low salt concentration (e.g., 100 mM NaCl) increased dry weight in arid-adapted accessions with respect to plants watered with control solution, whereas values registered for humid accessions decreased. Moreover, when watered with higher salt solutions (e.g., 400 mM NaCl), the relative dry weight reduction was lower in arid accessions than in humid-adapted accessions [48,49]. Shoot biomass and leaf area showed similar behaviour, although its values under no saline conditions were comparable [50]. This observation points to an obligate halophytic behaviour for arid-adapted plants, whereas humid-adapted accession of *C. maritima* performs as facultative halophytes. Different responses to salinity of accessions adapted to arid and humid regions rely on how these can activate biochemical strategies, which will be discussed below. Specific molecular pathways might be implicated in such adaptation to arid regions, although the knowledge in this regard is scarce. Proteomic comparison of salt-treated accessions adapted to low and high saline conditions reported the over-accumulation of 33 proteins upon salt treatment in both accessions, whereas there were 88 and 64 proteins accumulated specifically in arid-adapted and humid-adapted regions, respectively [51]. Thus, it seems likely that specific gene expression programmes are activated in accessions adapted to different bioclimate areas.

## 3. Disparate Responses of *Cakile maritima* to Salt Stress

In agriculture, toxicity is determined to be the concentration of a specific component of soils or any other media where plants grow, from which plant growth is declined. Both sodium and chlorine are toxic ions for plant development, and the threshold concentration from which toxicity occurs is species-specific. Soil salinity causes an increase in osmotic pressure that reduces the water available for plants and the accumulation in cells of Na^+^ cations that enter by non-selective channels, modifying the correct cellular osmotic pressure. The reproduction capability and plant vegetative growth are generally disturbed under saline conditions. The response to salinity of *C. maritima* has been studied in different biological processes, such as seed germination, overall vegetative growth, and reproduction fitness [52]. The following sections review different adaptation mechanisms described for *C. maritima*.

### 3.1. Anatomical and Morphological Adaptations of Cakile maritima

*Cakile maritima* is a succulent species that accumulates water in leaves [40]. Succulency is a common mechanism in halophytes that allows them to dilute toxic ions within tissues, reducing their toxicity. However, a relationship between succulency and salinity has not yet been described for *C. maritima*. Other morphological and anatomical adaptations of sea rockets under salt conditions have been reported. *Cakile maritima*’s overall growth is enhanced at low salt concentrations, whereas concentrations higher than 200 mM NaCl result in a reduction of biomass, explained by a reduction of leaf and node number, individual leaf surface area, and internode and root length [53]. Moreover, the stomata number in both the abaxial and adaxial sides of the leaves increases with salt concentration in a biphasic manner, showing the highest number of stomata per area at 300 mM NaCl. This anatomical adaptation correlates with a higher gas exchange ratio [53]. Moreover, *C. maritima* leaf dimorphism has been reported. There are indeed two different *C. maritima* morphotypes that differ in leaf shape, one characterised by entire lamina leaves and the other by pinnatifid leaves (Figure 1B); the pinnated leaf morphotype is more resistant to stress [38]. Further histological analysis of sea rocket leaves revealed specific structures related to salt responses, such as large thick-walled epidermal cells and prominent cuticles that prevent water loss, water-storage parenchyma, and hydathodes involved in the excretion of inner mineral compounds through guttation. Moreover, salt crystals were detected in the cell walls of vesicular epidermal cells [38]. Thus, sea rocket presents different morphological and anatomical adaptations involving stomata number, leaf shape, and anatomy, allowing this species to alleviate the toxicity caused by salt stress.

### 3.2. Cakile maritima Seed Formation and Germination under Saline Conditions

Seed germination is critical for seedling establishment and final plant development. Halophyte seeds are generally susceptible to high salinity, and the plants acquire resistance during vegetative growth [52]. Germination of *C. maritima* seeds is reduced upon the application of NaCl (200 mM or greater), although this effect is reversible, as shown in recovery experiments [52]. Therefore, salinity during germination does not affect seed viability but the germination process. Mild saline conditions delay seed germination, and the final germination rate is slightly reduced. This experimental set-up was used to identify proteins differentially accumulated in germinating seeds sown on mild (75 mM NaCl) saline media compared with control conditions. This proteomic approach led to the identification of proteins involved in glycolysis, amino acid metabolism, photosynthesis and protein folding that were differentially accumulated during germination [54]. Specifically, the mobilisation and degradation of several seed storage proteins, essential for proper seed germination and seedling establishment, were delayed in seeds sown on saline media. The exact reason is unknown, but one likely possibility is the reduced water availability under salinity due to osmotic pressure, which hinders hydrolytic processes [54]. Moreover, up to 12 proteins involved in different primary metabolism pathways, including photosynthesis, glycolysis, and glucogenesis, were identified as differentially accumulated under salt stress conditions. The fact that *C. maritima* seeds treated with NaCl show an overall normal but delayed protein accumulation pattern supports the idea that the seeds are still viable; however, germination is delayed by salt-induced dormancy [54].

Seed production is also affected by salinity during the development of mother plants. Seeds produced under strong salt stress conditions (i.e., 400 mM NaCl) are lighter and show a higher abortion rate [52,53] than the seeds of plants not subjected to high salinity. However, the exact biochemical mechanism that underlies these alterations during reproductive development has not yet been described, although it may be related, at least to a certain extent, to the impact of salt during vegetative development.

### 3.3. Cellular Osmotic Pressure Regulation in Cakile maritima

The strategies of a plant for dealing with the alterations of cytological osmotic pressure provoked by salt rely on *(i)* its capability to maintain low ion concentration within the cytoplasm, a mechanism that depends on ion extrusion, vacuolar transport, and retention activities, and *(ii)* the biosynthesis of organic solutes that maintain a proper cellular osmotic pressure [55]. *Cakile maritima* plants treated with increasing salt concentrations retain sodium and chloride in leaves, although the pattern registered for both ions is different; sodium is accumulated in a dose-dependent manner, whereas chloride accumulation increases at low salt concentrations and remains constant at higher concentrations. Accumulation of cations such as Ca^2+^, K^+^, and Mg^2+^ is lower than in plants grown under control conditions. These observations are explained at least in part by the higher activity registered by tonoplast and plasmatic membrane H^+^ ATPase pumps [56]. The sodium accumulation pattern in roots differs from that observed in leaves, being less pronounced [57] or even absent when plants were subjected to a high-salt shock [58]. A pharmacological approach using isolated cell cultures of *C. maritima* showed that sodium influx to the cytoplasm is partially reduced by applying two Non-Selective Cation Channel (NSCC) inhibitors. This accumulation resulted in cell death, although mortality of cells isolated from sea rocket was less pronounced than that observed for cells isolated from its related glycophyte *Arabidopsis thaliana* [58]. Moreover, an alternative SOS-like pathway implicated in Na^+^ extrusion from cells exists, and the expression of *CmSOS1*, *CmSOS2* and *CmSOS3* has been determined as constitutive in *C. maritima* cell culture [42].

Alternative mechanisms based on the biosynthesis of organic compounds that maintain cell osmotic pressure have also been reported during *C. maritima* response to salt stress. Both proline concentration [59] and polyphenol content [50] increased upon 20 and 28 days of salt treatment, respectively, in arid-adapted *C. maritima* accessions but not in plants adapted to humid bioclimate regions. Interestingly, proline overaccumulation was only detected upon high-salt (i.e., 400 mM NaCl) treatment [59], which indicates a specific biochemical response under extreme conditions different from the response to mild saline conditions (i.e., 100 mM NaCl) under which sea rocket growth is optimal. A metabolomics approach further demonstrated the distinct response to increasing salt doses. Low salt concentration induced a few amino acids and sugars biosynthesis, whereas high salt concentration resulted in a noticeable increase in the biosynthesis of amino acids and sugars and depletion of organic acid biosynthesis. Moreover, specific metabolites such as proline, GABA, and glycine were identified in plants treated under severe salt stress conditions [60]. Finally, no differences in α- or γ- tocopherol were detected in leaves of sea rocket plants cultivated in the presence of salt during the first 24 h of treatment, as observed in the related species *Arabidopsis thaliana* [61].

### 3.4. Antioxidant Responses of Cakile maritima to Stress

The negative effects of the cytotoxic ROS rapidly produced upon sodium perception can be alleviated by the activity of antioxidant enzymes or the biosynthesis of ROS scavenger molecules [31]. *Cakile maritima* activates an efficient antioxidant response that prevents oxidative stress within cells provoked by ROS. Activation of the antioxidative machinery occurs rapidly, within the first 24 h of salt exposure, and is maintained at least during 20 days of salt treatments. The biochemical responses of the halophyte *C. maritima* treated with 400 mM of NaCl and its related glycophyte *A. thaliana* watered with 100 mM NaCl solutions showed a different pattern, which explains the different adaptative mechanisms of these species to high salinity [62]. Accumulation of oxidative stress markers such as H_2_O_2_ and MDA peaked at four hours of treatment in sea rocket, whereas it constantly increased during the first 72 h in *Arabidopsis*. Accordingly, the activity of antioxidant enzymes (i.e., POD, CAT, and SOD) reached its maximum after four hours of treatment in *C. maritima*, whereas the activation of the *Arabidopsis* enzymes was not only delayed in time but also limited in their maximum values [62]. Moreover, a different antioxidant enzymatic response was detected upon mild and strong salt stress treatments. The activity of antioxidant enzymes such as catalase (CAT), peroxidase (POD), ascorbate peroxidase (APX), glutathione peroxidase (GR), and dehydroascorbate reductase (DHAR) increased under mild (100–200 mM NaCl) salt concentrations during long (20 days) time exposure. Only slight increases, however, were registered under high salt concentration (400 mM) at this time frame [63]. Accordingly, electrolyte leakage and concentrations of oxidative stress markers such as malondialdehyde (MDA) and hydrogen peroxide were higher upon 20 days of extreme salinity when compared with those registered for mild saline conditions [63]. These observations indicate that enzymatic detoxification of ROS is rapidly activated upon salt perception and point to a different enzymatic antioxidant response to different salt concentrations, which appears to be more efficient at low concentrations, in agreement with sea rocket’s performance under mild saline conditions. A more detailed study of SOD isozymes of *C. maritima* showed that, although Fe-SOD isozymes are the most prominent during overall plant development, CuZn-SOD isozymes are the ones that are primarily activated in response to severe salt stress [64]. All these data show that sea rocket can activate rapid and efficient antioxidant machinery in response to salt stress, based on several antioxidant enzymes, which maintain their increased activities during the salt treatment. This enzymatic response reduces oxidative damage and is a feature typical for halophytes since related glycophytes do not show such dynamics. Indeed, this behaviour depends not only on the species but also on the genotypes since arid-adapted accessions of *C. maritima* presented more active enzymatic antioxidant machinery than those from humid regions [48].

On the other hand, defence mechanisms based on the biosynthesis and accumulation of antioxidant compounds also contribute to salt tolerance in *C. maritima*. Leaves excised from sea rocket plants treated with different salt concentrations for 20 days accumulate increasing amounts of dehydroascorbate (DHAS) and reduced glutathione (GSH) in a dose-dependent manner [63]. An experiment performed during the first 72 h of salt treatment, however, detected glutathione accumulation, both in leaves and roots, whereas the glutathione redox stage was maintained constant during this period [57]; ascorbate accumulation was also detected in leaves and roots in this short time frame [57]. These data indicated that the biosynthesis of such antioxidant metabolites is rapidly activated upon salt perception, and their levels are maintained at least during the first 20 days of treatment. Interestingly, ascorbic acid accumulation was reduced when a saline shock using high salt concentration (400 mM NaCl) was applied to sea rocket plants [58].

**Figure 2:**
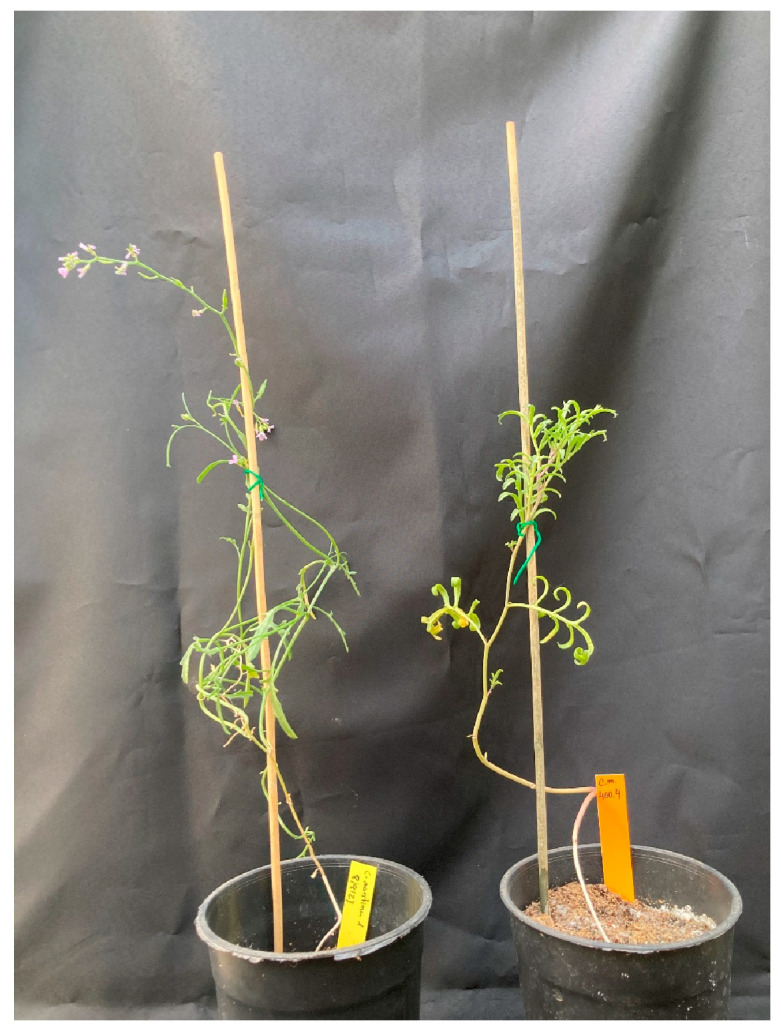
Plants of *Cakile maritima* watered with tap water (**left**) and 400 mM NaCl solution (**right**) for eight weeks.

In summary, apart from anatomical adaptations such as succulence, in response to high salinity conditions, *Cakile maritima* activates a series of biochemical defence mechanisms, primarily, as an includer species, the accumulation of toxic Na^+^ and Cl^-^ ions in the aboveground organs, while mitigating the cytotoxic effects of salt-induced ROS throughout the activation of antioxidant enzymes and the biosynthesis and accumulation of ROS-scavenging molecules. Although less studied, the transcriptional activation of SOS-based detoxification of sodium has also been reported [42]. It is difficult to estimate to what degree the regulation of ion transport and the activation of antioxidant systems, enzymatic and non-enzymatic, contribute to the observed behaviour of *C. maritima* in response to salt stress; most likely, both types of responses are relevant for salt tolerance in this species, allowing the plants to withstand salt concentrations up to 400 mM NaCl (Figure 2). Moreover, accessions adapted to different bioclimatic areas may differ in the efficiency of these general response mechanisms, and accessions adapted to arid habitats appear to be more salt-tolerant than those from more humid zones [48,49,59]. Since most of the reported information on *C. maritima* is limited to North African accessions, we cannot exclude the future identification of more or less efficient salt response mechanisms of this species in other climates and geographic areas.

### 3.5. Other Stresses

Certainly, most of the published studies related to the stress responses of *C. maritima* refer to salinity. However, the effects of other abiotic stresses, such as drought or nutrient starvation, have also been reported for sea rocket plants. Drought causes an overall growth arrest, although the plants’ recovery potential is very strong. Different physiological (e.g., stomatal conductance) and biochemical (e.g., glycine betaine and proline accumulation) responses are induced during drought stress [65]. Moreover, a proteomic study identified a significant alteration of proteins in plants subjected to drought compared with normally watered plants or plants recovered from the water stress treatment [66]. Potassium deprivation, on the other hand, resulted in oxidative stress in *C. maritima* plants, triggering a typical antioxidative response based on enhanced antioxidant enzyme activities [67], similar to that observed under high salinity conditions.

## 4. Economic Value of *Cakile maritima*

*Cakile maritima* is a widely spread wild species that plays a relevant ecological role in seashore environments [43,68]. Although the species is not cultivated commercially, individuals collected from their natural habitats have been traditionally used as edible or medicinal plants [69]. In the current climate change scenario, farmers, markets, and consumers are looking back to resilient traditional species and cultivars or stress-tolerant wild taxa such as halophytes [42], including *C. maritima*, as the basis of recovered or “new” cash crops better adapted to the challenging climatic conditions. In the following sections, some reported practical uses of *C. maritima* are described and discussed.

### 4.1. Cakile maritima as a Food Resource

Wild halophytes have been used as food resources for a long time [69]. A few of them, such as date (*Phoenix dactylifera* L.), sea fennel (*Crithmum maritimum* L.), or sea beet (*Beta vulgaris* subsp. *maritima*), are habitual in culinary cultures of different world areas [69]. The consumption of some other species, however, has been restricted to famine periods [69]. Recently, the search for new flavours, textures, and colours for the so-called gourmet cuisine, together with their beneficial nutritional features and salt tolerance, have made halophytes be considered for their commercial exploitation as food resources [69,70].

*Cakile maritima* is a species used for nutritional purposes. Nearly all organs are edible, and recipes for roots, young leaves, and stems, flowers, and fruits of *C. maritima* are available [69]. Young leaves and stems of sea rocket are good ingredients for fresh salads, whereas older leaves, due to their bitter flavour, are proposed to be used in soups or as a potherb, as well as for extracting flavouring additives or salsas. Similarly, fresh flowers are a gourmet ingredient for fresh salads and can add a slightly bitter flavour to soups. Roots can be ground to prepare flour. Finally, immature fruits can be eaten raw, and immature and completely mature fruits can be used as an oil source [69]. Nutritional analysis of sea rocket leaves revealed appropriate nutritional features for dietary purposes; the moisture content [68,71] and oxalic acid content [72] are particularly high in this species compared with other edible wild halophytes. Moreover, *C. maritima* accumulates elevated amounts of flavonoids and phenolic compounds, which confer a strong antioxidant activity [50,73], making this species attractive for its nutritional and healthy properties. Leaves, stems, and immature pods of sea rocket are commercially available as pickles in local markets.

Sea rocket seeds contain a high oil concentration comparable to those of its crop relative, rapeseed; therefore, it is considered an oleaginous species [74]. Fatty acid composition analysis, however, identified low amounts of healthy oleic acid, compared with olive oil, and a greater accumulation of erucic acid [74], which is dependent on the genotype [75] and the salt concentration [41,56]. These observations urge us to be careful when considering sea rocket seeds to obtain oil for human and animal consumption since erucic acid seems to trigger fat deposition in heart muscle and myocardial lesions [76]. Thus, searching for natural genetic variants with low or no erucic acid can make the sea rocket an ideal species for the obtention of edible oil. For instance, erucic acid in extracts of Egypt sea rocket accessions is under the detection limit, whereas oleic acid is the most abundant fatty acid detected [77]. Moreover, oil quality differs from accessions adapted to different bioclimate regions within the same geographical regions [47,75]. On the other hand, the development of breeding programmes towards obtaining erucic acid-free *C. maritima* cultivars, as it was previously done in rapeseed [78], is a suitable alternative.

**Figure 3:**
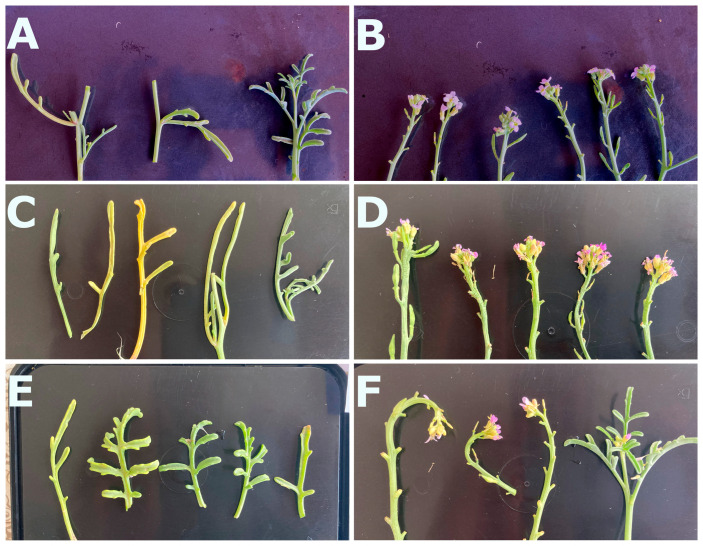
General aspects of leaves (**A**,**C**,**E**) and flowers (**B**,**D**,**F**) excised from adult *Cakile maritima* plants growing under natural conditions. (**A**,**B**) Leaves and flowers at day 0. (**C**,**D**) Leaves and flowers at day 10, stored at 4 °C under dry conditions. (**E**,**F**) Leaves and flowers at day 10, stored at 4 °C under humid conditions.

Therefore, sea rocket plants are a suitable food source due to their original flavour, nutritional properties, and oil content, with several manners of presenting, making *C. maritima* an ideal species for innovative cuisine. Moreover, we have observed a relatively long conservation time in basic fridge conditions (4 °C), either open (dry) or contained in a closed recipient (humid), which is an advantage for post-harvest commercial purposes. We observed that the crunchy texture and turgor of leaves and stems were maintained for 15 days of storage at 4 °C, although the leaf colour started to shift from green to yellow (Figure 3). Upon 28 days of storage, the turgor and colour of leaves were lost, although the flavour was comparable to the first days upon harvesting (Figure 3). These are only visual observations; whether this is accompanied by the conservation of *C. maritima*’s nutritional and culinary properties is still to be studied. In any case, it seems likely that sea rocket is not a perishable species, which is a valuable quality for food markets.

### 4.2. Medical Uses of Cakile maritima

Besides being used as food, sea rockets are traditional medicinal plants due to their several medical applications, such as diuretics, antiscorbutics, appetising, digestive, and purgative [40,79]. *Cakile maritima*, similar to other Brassicaceae, synthesises glucosinolates and camalexin [77], which are bioactive compounds with antibiotic properties [80]. Indeed, its antimicrobial activity against several microorganisms has been reported [73,81]. Moreover, *C. maritima* extracts have been used as a chemotherapeutic drug against scurvy due to their ability to accumulate elevated concentrations of vitamin C [79]. Finally, transcriptional analysis of genes involved in the inflammatory response of isolated human cells showed that extracts of all organs tested (leaves, stems, seeds, and flowers) alleviated the inflammatory response; thus, sea rocket extracts have in vitro anti-inflammatory activity [81]. The same authors showed that sea rocket extracts inhibited the proliferative growth of tumour cells in a dose-dependent manner, with no cytotoxic effect in tumour or control cells [81]. All these reports demonstrate that *C. maritima* is a plant species suitable for the search for new active compounds with pharmacological applications.

### 4.3. Environmental Benefits of Cakile maritima

The adaptation to extreme environments of halophytes makes such species suitable for phytoremediation of soils and water since they have developed specific strategies, including the accumulation of toxic compounds. On the one hand, due to their natural capability to uptake and accumulate Na^+^ and Cl^−^ within their aerial parts, halophytes, in particular includer species such as *C. maritima*, are suitable for desalination and recovery of salinised cultivated areas, either by intercropping or in crop rotation programmes [82]. In this sense, it has emerged the so-called circular halophyte mixed farming (CHMF; Ref. [83]), is a farming approach that advocates for the cultivation of halophytes and further harvest in saline areas to recover soil quality for agronomic uses. This strategy is known as haloremediation, and several halophytes have already been assayed for this purpose [84].

On the other hand, *C. maritima* presents relatively high levels of glutathione-S-transferase activity, an enzyme that directly participates in the biotransformation of xenobiotic compounds [85]. The potential use of *C. maritima* as a phytoremediator has been assessed by its capability to uptake from soils radionuclides naturally occurring in the environment, uranium and thorium, and accumulate them in the aerial parts of the plant, although its phytoremediation efficiency is significantly lower than for other species tested [86]. Nevertheless, this may depend on the specific toxic heavy metal. For example, a comparative analysis of *C. maritima* and *Brassica juncea* indicated that the former is a more efficient phytoremediator for lanthanum (La) [87] and less efficient for barium (Ba) [88]. Moreover, physiological comparations of *C. maritima* plants grown in control versus cadmium (Cd) contaminated soils revealed a high tolerance and Cd accumulation in stems of sea rocket [89]. Indeed, the Cd translocation factor is higher for *C. maritima* than for *B. juncea*, a species traditionally used as a Cd detoxification model [89].

Apart from the sea rocket’s potential for desalination and heavy metal phytoremediation, its deep root system with numerous lateral roots makes this species important for soil fixation, for example, in the coastal dunes, providing essential ecosystem services in its natural habitats [90].

### 4.4. Other Uses of Cakile maritima

The main practical uses of sea rocket plants have been described in the previous sections, but *C. maritima*‘s chemical properties make it also attractive for different industries. On the one hand, its high content of flavonoids and phenolic compounds, which confer a strong antioxidant activity [73], is an interesting feature for the cosmetics industry and dermatological care [79]. On the other hand, erucic acid (EA)-rich oils are valuable for their industrial applications. Erucic acid (C22:1) is a long-chain monounsaturated fatty acid that is highly hydrophobic and water-resistant. For this reason, such an oil is used as a lubricant, for plastic production, and as a rubber additive. EA-rich oils can be produced by plants, mostly from the Brassicaceae family, and by some fish species. Currently, high-EA rapeseed (HEAR) varieties, non-suitable for human consumption, are the primary source of erucic acid, and there are numerous breeding programmes based on GMO approaches aimed at obtaining oil with higher and more available EA, as well as with less EA competitors [91]. This fact shows the economic importance of erucic acid due to its industrial applications. In this scenario, sea rocket could be an alternative source for EA-enriched oils, with the additional benefit of its lower requirements for cultivation in the context of “biosaline agriculture”: in saline soils and irrigated with saline water, condition unsuitable for glycophytic oilseed crops. In any case, more research is needed to obtain *C. maritima* EA-rich lines and develop oil extraction methods from *C. maritima* seeds that are as efficient as those established for seeds of conventional oilseed species such as rapeseed.

## 5. Conclusions and Future Perspectives

*Cakile maritima* is a relatively small and fast-growing species that can produce many seeds (ca. 10,000) per growing cycle [41,42]. These characteristics, together with its halophytic behaviour, make the sea rocket an ideal model species for studying plant responses to salt. Several studies have been reported that contribute to uncovering the biochemical mechanisms that allow sea rocket to alleviate the adverse effects of salinity during vegetative growth. However, there are still some unexplored questions, such as the accumulation of osmotic regulatory metabolites other than proline, GABA and glycine, the SOS-dependent pathway to expulse sodium from the cytosol, or the plausible different mechanisms of adaptation and response to mild and intense saline conditions used by *C. maritima*. Finally, almost all reported work has focused on *C. maritima*‘s response to salinity during vegetative growth, but the response during flowering and reproductive development is practically unexplored, other than seed production and germination experiments [52,56].

On the other hand, *C. maritima* is a good candidate amongst halophytes for the so-called biosaline agriculture due to its different uses as a potential cash crop, as a food source, a medicinal plant, or for the extraction of compounds with industrial applications, as well as for phytoremediation/haloremediation of contaminated soils and water. The high genetic variability of this species, due to its facility for outcrossing and dispersal of seeds [40], makes its potential agronomic use challenging. However, this should be taken as an opportunity for breeders since high genetic variability is preferred for searching for variants adapted to specific climate conditions and uses. In this sense, and taking into account that sea rocket belongs to the same family as the highly embryogenic *Brassica napus* [92], approaches directed to the obtention of double haploid (DH) lines through androgenesis is a proper starting point for *C. maritima* breeding.

Finally, the commercial cultivation of promising halophytes, including *C. maritima,* can contribute to socio-economic development in marginal areas by providing livelihood opportunities, reducing rural poverty, and promoting resilience to climate change impacts.

## Data Availability

Data are contained within the article.
